# Mortality risk and burden associated with non-optimum temperatures in Puerto Rico

**DOI:** 10.1088/1748-9326/ae013e

**Published:** 2025-09-09

**Authors:** Francisco Díaz-Collado, Lingzhi Chu, Daniel Carrión, Pablo A Méndez-Lázaro, Kai Chen

**Affiliations:** 1 Department of Environmental Health Sciences, Yale School of Public Health, New Haven, CT, United States of America; 2 Yale Center on Climate Change and Health, Yale School of Public Health, New Haven, CT, United States of America; 3 Environmental Health Department, Graduate School of Public Health, University of Puerto Rico, San Juan, PR, United States of America

**Keywords:** heat, mortality, Puerto Rico, Caribbean, small island developing states (SIDS)

## Abstract

The effects of a changing climate are already evident in Caribbean small island developing states (SIDS) like Puerto Rico, where heat episodes have become more frequent. Despite reports of increasing heat-related death rates, robust epidemiological evidence on the health impacts of high temperatures, as well as the effects of low temperatures, remains scarce, particularly outside of urban settlements in Caribbean SIDS. In this study, we conducted a case time-series study on municipality-level mortality and temperature in Puerto Rico from 2015–2023. We modeled the relationship between daily mortality count and mean temperature using a conditional quasi-Poisson regression, combined with a distributed lag non-linear model (dlnm) with a 21 d lag, adjusting for relative humidity, seasonality, and day of the week. We estimated the minimum mortality temperature (MMT)—the optimal temperature associated with the lowest mortality risk—and calculated the relative risk associated with extreme low and high temperature, defined as the 2.5th and 97.5th percentiles of daily temperature. Additionally, we estimated the municipality- and island-level excess mortality fractions attributable to both low and high temperatures, relative to MMT. Our findings indicate that exposure to non-optimum temperatures (both low and high temperatures) is significantly associated with increased mortality risk. Specifically, extreme low temperature was associated with a 1.23 (95% CI: 1.07–1.40) times risk of all-cause mortality, while extreme high temperature was associated with a 1.16 (95% CI: 1.05–1.27) times risk. We estimated that temperature-related mortality accounted for 3.88% of the total 280 568 deaths (95% eCI: 3.39%–4.29%), with low temperatures contributing 2.02% (95% eCI: 1.69%–2.32%) and high temperatures contributing 1.86% (95% eCI: 1.35%–2.35%). Furthermore, we found substantial spatial variability in temperature-related mortality burdens across municipalities. Our study identifies the vulnerable municipalities to temperature-related deaths in Puerto Rico, providing evidence to inform municipality-specific climate adaptation and mitigation strategies.

## 1. Introduction

Extensive research demonstrates that the relationship between temperature and mortality follows a J- or U-shape curve, where mortality risk increases at both low and high temperatures [1, 2]. Between 2000 and 2019, an estimated 9.43% of excess deaths globally were associated with exposure to non-optimal (both low and high) temperatures [3]. However, most of this evidence originates from high-resource cities with robust systems for monitoring environmental exposure and health outcomes [3–5]. Expanding research to underrepresented regions is crucial, as substantial regional variability in climate, population vulnerability, and adaptive capacity limit the generalizability of existing findings across diverse settings. This heterogeneity underscores the need for developing locally relevant technologies and policies to address the health impacts of climate change.

While some argue that climate change may pose benefits in certain regions, a warming climate is expected to have predominantly detrimental effects particularly in small islands developing states and territories (SIDS) [6–9]. Despite their high vulnerability, SIDS are largely underrepresented in temperature-related mortality research. Previous research found a potential increased mortality risk associated with both heat and cold temperatures in a few Caribbean SIDS [10, 11]. However, these studies were confined to urban areas, providing an incomplete view and reducing applicability across both urban and rural areas in Caribbean SIDS. Moreover, previous studies are limited by not accounting for variations in socioeconomic conditions, demographic profiles, and climates patterns outside of urban settings [10–13]. However, efforts to address this knowledge gap are hindered by barriers in data equity in Caribbean SIDS, such as the limited availability and spatial and temporal resolution of environmental and health data due to inadequate monitoring systems or restricted access.

As a US territory, Puerto Rico is an Associate Member of the United Nations Commission for SIDS. Puerto Rico—an archipelago of 3.2 million people—currently ranks as 33rd most populous US states and territories. Its capital city, San Juan, is the largest city examined in previous temperature epidemiology studies of Caribbean SIDS [3, 10]. However, most research has examined the impacts of heat rather than the full range of non-optimum temperatures across different geographic areas [14–16]. The health effects of moderate and extreme temperatures remain poorly understood in Puerto Rico. Since 1950, average temperatures have risen by 2 °F (∼1.1 °C), with minimum temperature increasing rapidly, especially at low elevations [17]. A previous study found an elevated risk of mortality—primarily from stroke and cardiovascular diseases—in the municipalities of San Juan and Bayamón, particularly over the summers of 2012 and 2013 [15]. At the time of its publication (2018), the summer of 2012 was the warmest on record in San Juan, with June 2012 also holding the record as the hottest individual month [18]. However, recent data show that summer 2024 has now surpassed 2012 as the warmest summer season, with its months ranking among the hottest on record. September 2024 now holds the highest average monthly temperature on record, followed by June 2024, August 2024, September 2023, and July 2024 [19, 20]. In June 2024, meteorologists issued the first heat advisory covering all 78 municipalities of the island [21], underscoring the urgent need for widespread research on these health impacts in the recent decade with rapid global warming, particularly in rural areas.

Considering the spatial variation of climatic characteristics in Puerto Rico [22], using a city-level exposure assessment fails to capture the substantial heterogeneities across municipalities. Therefore, a comprehensive island-wide analysis on the municipality level is essential to fully capture the health risks posed by a changing climate in Puerto Rico. However, the traditional time-series approach has challenges in analyzing small areas, where a small sample size may not provide sufficient statistical power. To tackle these challenges, we used a recently proposed design—the case time-series design, which integrates the modeling framework of a time-series analysis with the self-matched structure of case-only studies [23]. This design enhances the spatiotemporal resolution of analysis, offering numerous advantages in ensuring sufficient statistical power for small areas, being compatible with granular exposure assessment, allowing for heterogeneities across locations, and reducing the possibility for ecological fallacy by avoiding larger-scale data aggregation [24]. Leveraging the novel case time-series design, this study aimed to quantify the burden of non-optimal temperatures on all-cause mortality across municipalities in Puerto Rico.

## 2. Methods

### 2.1. Data collection

Daily all-cause mortality records from 2015 to 2023 across all 78 municipalities in Puerto Rico were obtained from the Vital Statistics Office of the Puerto Rico Department of Health. These records included sociodemographic characteristics such as age, sex, marital status, and education. Additionally, for Puerto Rico and its municipalities, we gathered annual population estimates and six socioeconomic indicators from the U.S. Census Bureau [25, 26]: (a) population by age, (b) population with an educational attainment below than 12th grade, (c) population without health insurance, (d) population living below the poverty line, (e) population living with a disability, and (f) population density.

We define the exposure as the meteorological conditions experienced in the municipality of residence reported on death records. Meteorological data—including daily vapor pressure, maximum and minimum temperature—were obtained from the Daily Surface Weather and Climatological Summaries (Daymet), which provides high-resolution spatial data that accurately predicts ground meteorological data over North America, Hawaii, and Puerto Rico [27, 28]. To generate daily municipality-level weather parameters from 2015 to 2023, we overlaid the 1 × 1 km gridded estimates with Puerto Rico’s municipal boundaries and spatially averaged grid cells within each municipality. We calculated the daily mean temperature as the average of the daily maximum and minimum temperatures. Relative humidity was calculated based on an established formula, following recommendations for its use in epidemiological studies [29].

### 2.2. Statistical analysis

We utilized a single-stage, case time-series design to assess the association between temperature and all-cause mortality across all municipalities, while also enabling municipality-specific effect estimates. The relationship between temperature-mortality was modeled using a conditional quasi-Poisson regression model [30] combined with a distributed lag non-linear model (dlnm), implemented by the *gnm* package in R [31]. The general model structure is presented below:

\begin{align*} &amp; {\text{Log }}\left( {E{ }({Y_{i,t}})} \right)))\nonumber\\ &amp; \quad= {\alpha _i}_{(k)} + { }\beta {\text{ Cb tem}}{{\text{p}}_{i,t}} + {\text{ns}}\left( {{\text{r}}{{\text{h}}_{i,t}},{\text{ df}} = 4} \right)\nonumber\\ &amp; \qquad + {\text{ns}}\left( {{\text{date}},{\text{ df}} = {6^*}{\text{ number of years}}} \right) \nonumber\\ &amp; \qquad + \gamma \,{\text{dow + offset}}\left( {{\text{log}}\left( {{\text{Po}}{{\text{p}}_{(i)}}} \right)} \right)\end{align*}

where *t* denotes date, *i* denotes respective municipalities (*i* = 1, 2, …, 78), and *k* denotes stratum of municipality-year-month [24]. In this fixed-effects framework, the stratum-specific intercepts *α_i_*_(*k*)_ capture variation in baseline mortality risk across municipalities and absorb the influence of all time-invariant confounders [23, 24]. This design inherently controls for potential confounding from such factors, as they are conditioned out within the regression structure [23, 32]. *E(Y)* represents the daily count of all-cause mortality. Exposure to temperature, modeled as Cb temp was captured using a cross-basis for mean temperature. We applied a natural cubic spline (ns) with knots placed at the 10th, 75th, and 90th percentiles of the island-wide temperature distribution. To capture the lag-response association, we placed three equally spaced internal knots on the logarithmic scale, with a maximum lag of 21 d. This structure allows for the assessment of potential delayed effects and mortality displacement. These methodological choices were adapted from previous research and literature [4]. We adjusted for relative humidity (rh) using a natural cubic spline (ns) with 4 degrees of freedom (df), for seasonality and long-term trends using a ns for the date (df = 6 per year), and for weekly patterns using an indicator for the day of the week (dow). Additionally, we incorporated the logarithm of the annual municipality population as an offset term. We estimated the relative risk (RR) as the rate ratio of mortality for extreme low and high temperature, defined as the 2.5th and 97.5th percentiles of the temperature distribution, respectively. These percentiles were chosen to capture more severe and less frequent temperature extremes, which may be more relevant for public health planning in a warming climate.

We explored effect modification at both the individual and municipal levels. For individual sociodemographic factors (i.e. sex, age, marital status, and education), we stratified the analysis by subgroups and assessed heterogeneity with two-sample *z*-tests [33]. For municipal socio-economic factors—percentage population aged 65 and over, percentage of low education attainment, percentage of uninsured, percentage of poverty, percentage of disability, and population density percentile—we extended the main model using the two-way effect modification approach for binary variables [34]. Each indicator was categorized as low or high based on the median value across all municipalities. Municipalities were also classified according to their average relative humidity and urbanicity, defined as population density ⩾1000 mi^2^ and population size ⩾50 000 [35]. Statistical significance of these interactions was evaluated with the likelihood ratio test.

We estimated excess mortality counts (EMN) and fractions (EMF) associated with low and high temperatures. For each municipality and day, the EMN was calculated using the pooled exposure-response function corresponding to the daily temperature, following the approach described in previous studies [4, 36]. This approach assumes a constant temperature-mortality relationship across all municipalities. The total EMN associated with non-optimum temperatures was calculated by summing daily contributions over the study period, while the EMF was computed as the ratio of EMN to total mortality. We conducted this process at both the municipality and the island level. To distinguish between the impacts of low and high temperatures, we separated these estimates into days with temperatures below or above the minimum mortality temperature (MMT)—temperature at which the mortality risk is lowest—and then aggregated the contributions and divided by total mortality. We estimated empirical confidence intervals (95% eCI) through 1000 Monte Carlo simulations.

To test the robustness of our results, we conducted several sensitivity analyses. These included (a) redefining extreme temperatures using alternative thresholds of the temperature distribution: the 1st and 5th percentiles for low temperature, and the 95th and 99th percentiles for high temperature; (b) modifying the df per year (7 and 8 df); (c) testing alternative maximum lags for temperature exposure (7 and 14 d); (d) excluding relative humidity as a covariate; and (e) using maximum and minimum temperature as alternative exposure variables. All analyses were performed using R software (version 4.4.1) with the *dlnm, gnm*, and *splines* packages.

## 3. Results

### 3.1. Descriptive analysis

**Figure 1.**
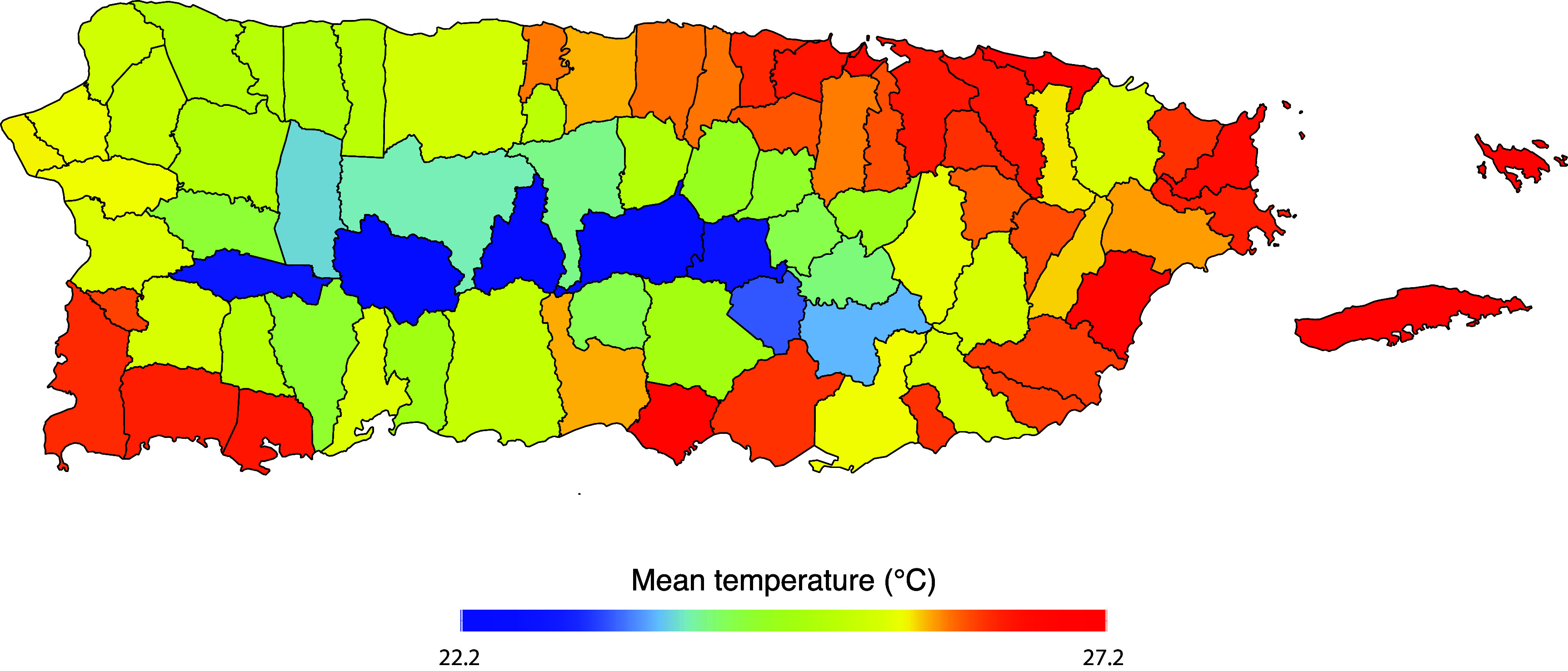
Mean daily temperature (°C) over 2015–2023 across the municipalities of Puerto Rico.

During the study period (2015–2023), a total of 280 568 deaths were recorded (table 1). Of the total deaths, 46% were male, and 54% were female. Deaths from the elderly population (aged 65 and above) accounted for 77%, while deaths from the younger population (under 65 years) accounted for 23%. Marital status was categorized as single (21%), married (32%), divorced (15%), and widowed (30%). About half of death cases were either without (51%) or with (49%) a high school diploma. The average mean temperature in Puerto Rico was 26.3 °C (SD = 1.85), and the average relative humidity was 73% (SD = 5%). Figure 1 illustrates the mean temperature distribution across municipalities, ranging from 23.1 °C to 27.8 °C. Warmer temperatures were observed in coastal and island municipalities, whereas cooler temperatures occurred in municipalities located in the central region.

**Table 1.** Summary statistics of daily mortality and weather conditions in Puerto Rico from 2015–2023.

Summary statistics	N (%)
**Number of deaths**	280 568
**Sex**	
Male	151 726 (46%)
Female	128 831 (54%)
Missing	11 (<1%)
**Age**	
Elder (⩾65)	216 288 (77%)
Younger (<65)	64 264 (23%)
Missing	16 (<1%)
**Marital**	
Single	58 248 (21%)
Married	90 158 (32%)
Divorced	42 118 (15%)
Widowed	84 677 (30%)
Missing	5,367 (2%)
**Education**	
No High School	142 696 (51%)
High School	132 217 (47%)
Missing	5655 (2%)
**Weather [mean (SD)]**	
Mean temperature (°C)	26.26 (1.85)
Mean relative humidity (%)	0.73 (0.05)

### 3.2. Association between temperature and mortality

**Figure 2.**
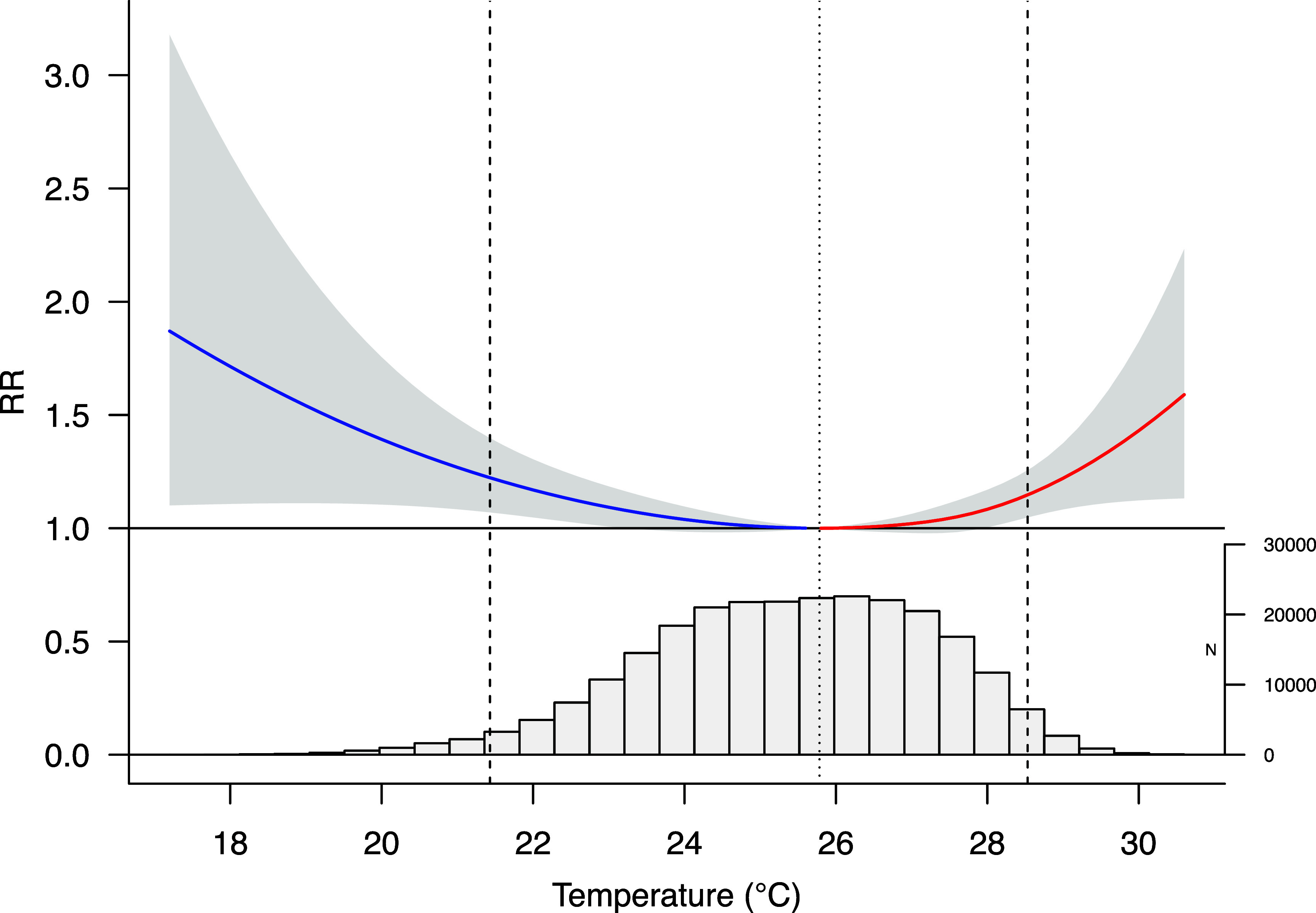
Exposure-response curve of the relationship between non-optimum temperature and mortality. Dashed lines indicate 2.5th and 97.5th percentiles of the temperature distribution. Dotted line marks the MMT.

Figure 2 shows the overall cumulative exposure-response relationship between temperature and all-cause mortality, which follows a ‘U’-shaped pattern, indicating that both low and high temperatures are associated with increased mortality. Mortality risk was significantly elevated for both extreme low temperature (RR = 1.23, 95% CI: 1.07–1.40) and extreme high temperature (RR = 1.16, 95% CI: 1.05–1.27).

We found that effect estimates for extreme temperatures remained consistent when increasing the df per year or removing the relative humidity adjustment from the model (table 2). However, estimates were sensitive when reducing the lag of the exposure to 7 and 14 d. The effects of low temperature lost its significance at lag = 7, while the effects of high temperature remained significant. Additionally, while using the daily minimum temperature as the exposure, only the impacts of high temperature stayed significant, whereas using the daily maximum temperature, only the impacts of low temperature stayed significant. When adjusting the threshold of extreme low and high temperature to the 1st, 5th, 95th and 99th percentile of the temperature distribution, associations remained robust for the overall estimates (tables S1 and S2).

**Table 2.** Point estimates of temperature-related mortality associated with extreme low and high temperatures.

Adjustment	RR (low temp.)	RR (high temp.)
df = 7	**1.22 (1.06, 1.41)**	**1.15 (1.04, 1.27)**
df = 8	**1.23 (1.07, 1.42)**	**1.12 (1.01, 1.24)**
lag = 7	1.06 (0.98, 1.15)	**1.09 (1.03, 1.15)**
lag = 14	**1.13 (1.02, 1.26)**	**1.12 (1.04, 1.21)**
Tmean^a^	**1.24 (1.08, 1.42)**	**1.14 (1.04, 1.25)**
Tmin	1.01 (0.91, 1.12)	**1.30 (1.15, 1.47)**
Tmax	**1.23 (1.10, 1.36)**	1.08 (1.00, 1.17)

^a^ Adjustment only considers mean temperature and no relative humidity. **Bolded** values indicate significant results.

### 3.3. Effect modification by sociodemographic and socio-economic indicators

Effect modification by sociodemographic factors is illustrated in figure S1. Extreme low temperature was significantly associated with increased mortality among females (RR = 1.36, 95% CI: 1.11–1.66), the elderly population (RR = 1.29, 95% CI: 1.11–1.51), singles (RR = 1.35, 95% CI: 1.01–1.82), widowed individuals (RR = 1.38, 95% CI: 1.08–1.76), and those without a high school diploma (RR = 1.23, 95% CI: 1.02–1.47). Similarly, extreme high temperature was significantly associated with increased mortality among females (RR = 1.22, 95% CI: 1.07–1.40), the elderly population (RR = 1.16, 95% CI: 1.04–1.29), married individuals (RR = 1.22, 95% CI: 1.04–1.45), and those without a high school diploma (RR = 1.20, 95% CI: 1.04–1.37). We did not find significant differences when conducting the two-sample *z*-tests between the sociodemographic subgroups. Additional RRs at the 1st and 99th percentiles are shown in figure S2, and RRs at the 5th and 95th percentiles appear in figure S3.

**Figure 3.**
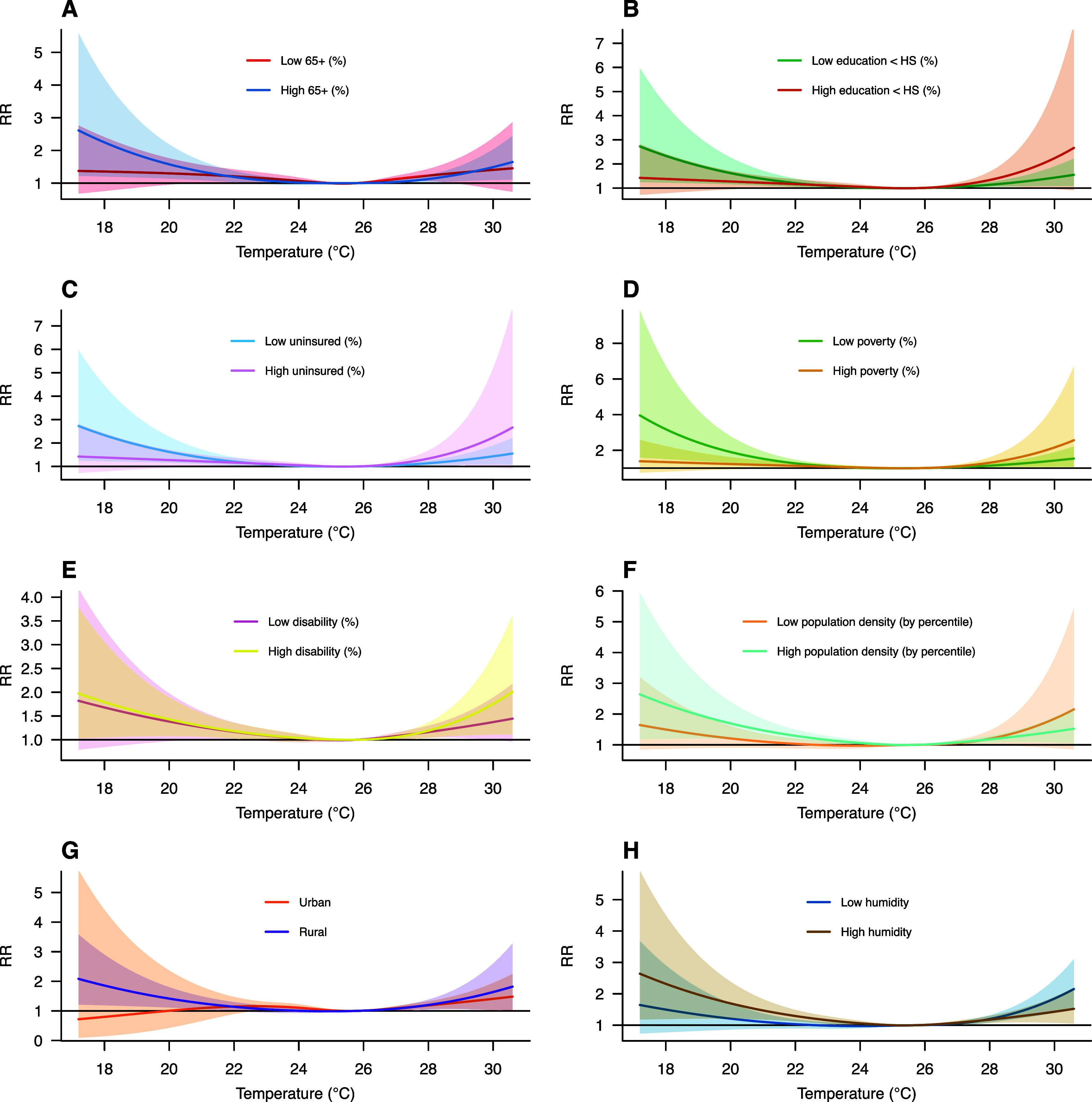
Exposure-response curve of the relationship between non-optimum temperature and mortality for low and high (defined by the median): (A) percentage of population aged 65 and over, (B) percentage of population with education less than HS (High School), (C) percentage of population without health insurance, (D) percentage of population below the poverty line, (E) percentage of population living with a disability, (F) percentile of population density, (G) urbanicity and (H) humidity.

Municipal socio-economic and humidity indicators are mapped in figure S4. Their modifying effects on the temperature-mortality relationship are presented in figure 3. All exposure-response curves preserved the ‘U’-shaped pattern observed in the main model. While the temperature-mortality relationships remained generally consistent across low and high categories, slight shifts in curve shape and confidence interval width occurred near temperature extremes. However, none of the seven indicators significantly modified the temperature-mortality relationship, as all yielded *p*-values > 0.05 in the likelihood ratio test.

### 3.4. Mortality burden associated with non-optimum temperatures

**Figure 4.**
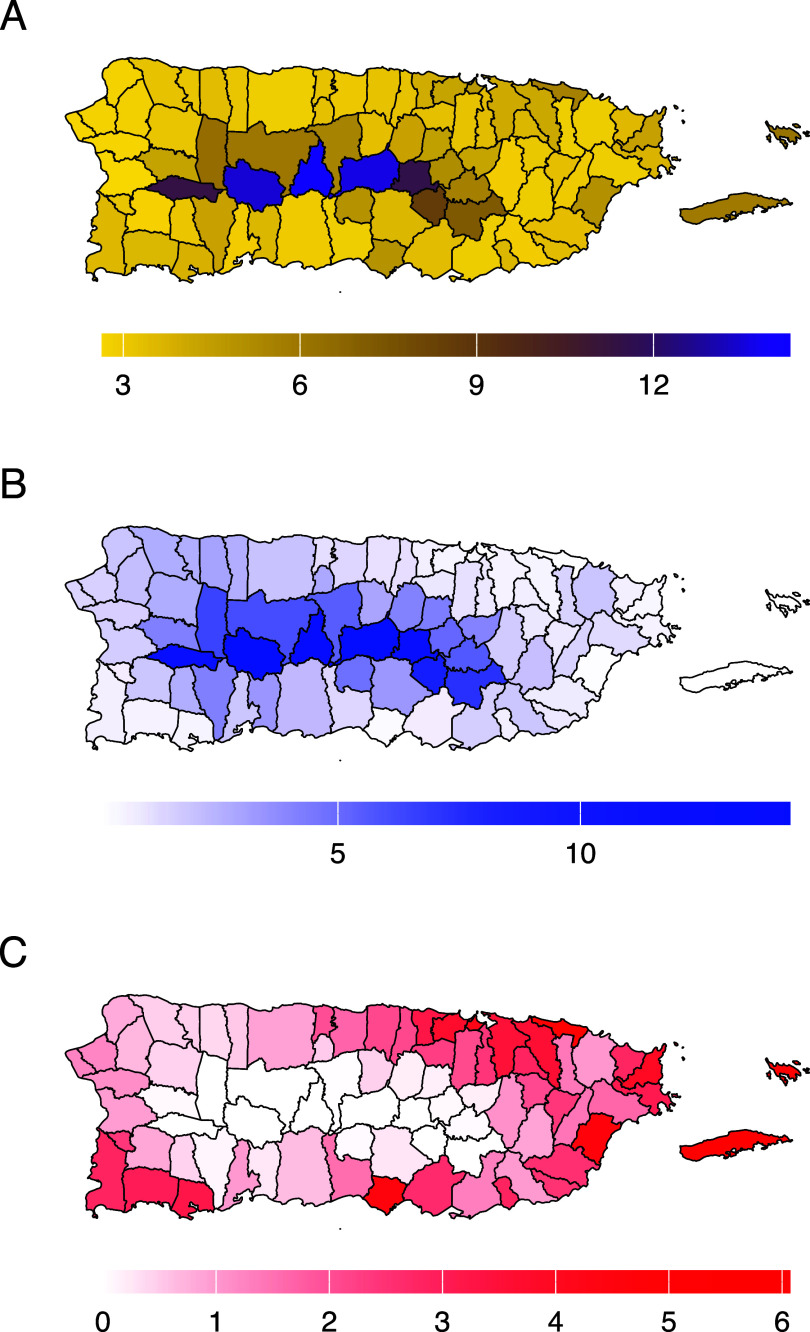
Percentage of excess death (EMF) by municipality. (A) EMF associated with non-optimum temperatures. (B) EMF associated with low temperatures. (C) EMF associated with high temperatures.

The EMF associated with non-optimum temperatures was 3.88% (95% eCI: 3.39–4.29), with low temperatures accounting for 2.02% (95% eCI: 1.69–2.32) of EMF and high temperatures accounting for 1.86% (95% eCI: 1.35–2.35). Figure 4 presents the EMF associated with non-optimum temperatures across municipalities. All three EMF exhibited high variability across municipalities. The EMF associated with non-optimum temperatures (figure 4(A)) and low temperatures (figure 4(B)) were higher towards the central areas of the island. In contrast, the EMF associated with high temperatures increased in coastal and island municipalities (figure 4(C)).

## 4. Discussion

To the best of our knowledge, this study represents the first effort to estimate the association between temperature and mortality across all municipalities in Puerto Rico. Notably, it is also the first to report significant results using a case time-series design in Puerto Rico and, more broadly, across Caribbean SIDS, where traditional time-series approaches have often failed to detect robust associations. Our results are consistent with previous studies that have found a significant increase in mortality risk associated with non-optimum temperature, as well as similar shapes of the association observed in other locations across the globe, Central and South America, and tropical climates [3, 4, 10, 37–40].

Specifically, our findings indicate that extreme low and high temperatures are associated with a 1.23% and 1.16% increased risk in mortality, respectively. Studies examining the relationship of mortality and non-optimum temperatures identified a moderate increased risk of mortality in San Juan associated with both cold and heat temperatures, but lacked statistical significance [10, 11]. By expanding our analysis beyond the island’s capital, we detected significant associations between temperature exposure and mortality, comparable to findings from other tropical regions in Latin America and Asia. Pooled estimates from 326 Latin American cities suggest RRs of 1.192 for cold and 1.057 for heat. Similarly, in Pune, India—a subtropical city with a mean temperature of 24.6 °C—the estimated mortality risk associated with cold and heat is 1.25 and 1.11, respectively [39].

Exposure to non-optimum temperatures accounted for an estimated 3.88% of excess deaths, with low temperatures contributing the largest burden (2.02%) and high temperatures accounting for 1.86%. This finding aligns with research from both temperate and tropical regions, despite the narrower range of temperature in tropical settings [3, 4, 10, 37, 39]. Due to the increased granularity our study, we identified municipality differences of temperature-related mortality across Puerto Rico (figure 4). Mid-center municipalities experienced a greater mortality burden from cool temperatures, while coastal and island municipalities had a greater mortality burden from warm temperatures. These differences in burden largely reflect differences in mean temperature exposure (figure 1), as the same pooled exposure-response function was applied to all municipalities. Urban settlements in Puerto Rico are located in coastal municipalities, particularly in the metropolitan northeast, where the urban heat island effect has been documented [41]. The southern region of Puerto Rico has a drier climate due to a rain shadow effect caused by easterly trade winds [22]. In contrast, mid-center municipalities experience cooler temperatures due to their mountainous characteristics and higher elevations [22]. These climatic and geographic characteristics likely explain the observed spatial patterns in temperature-related mortality.

Our findings on the increased mortality risks of non-optimal temperatures offer important geographical insights. Over the past decades, Caribbean SIDS were regarded as one of the highest rates of increase of heat-related deaths [6]. However, empirical evidence linking temperature exposure to mortality in the region remains limited. Previous research on San Juan and Bayamón aligns with the broader literature on the impacts of heat on mortality, particularly due to cardiovascular diseases [15, 42]. However, cause-specific health impacts beyond those two municipalities continue to be unexplored. Despite the consistently warming temperatures, modest reductions from typical temperature ranges can significantly impact health. Due to acclimatization, populations accustomed to warm temperature may experience adverse health effects when exposed to cooler conditions that, while mild in absolute terms, are unusual for the region. As a result, lower-than-normal temperatures can increase mortality risk—a phenomenon observed in previous multi-country studies [3, 4].

Our analysis did not observe statistically significant effect heterogeneity by individual sociodemographic factors or effect modification by municipal socio-economic indicators. However, we found an increased mortality risk among specific subgroups. Mortality risk associated with extreme low temperature increased significantly among females, the elderly, singles, the widowed, and those without a high school diploma. Similarly, mortality risk associated with extreme high temperature increased significantly among females, the elderly, married individuals, the widowed and those without high school diploma. At the municipal level, we noted slight differences in the exposure-response curves, but none of the socio-economic indicators significantly modified the temperature-mortality association. Unlike other regions in which low proportion of the elderly, urbanization, humidity, and healthcare seem to significantly affect these associations [43–45]. While the mechanisms behind these patterns remain unclear, previous studies suggest that sociodemographic and socio-economic factors may shape exposure profiles and influence vulnerability. For instance, females and older adults may be more vulnerable to temperature related stress due to physiological differences in thermoregulation and adaptative capacity [46, 47]. Social factors such as isolation and low income—more prevalent among certain subgroups—may limit help-seeking behavior during health crises, contribute to prolonged exposure and reduced resources to mitigate temperature-related stress [46, 48, 49]. Additionally, in Puerto Rico, agriculture, farming, and construction industries are among the top ten sectors of recruiting individuals without a college degree [50–52]. Importantly, only 35% of agriculture workers report holding a bachelor’s degree [53]. This suggests that lower education levels may be an important indicator of occupational exposure through physically demanding outdoor jobs, as well as limited resources to mitigate temperature-related stress [46, 49].

Nighttime exposure to heat may play a crucial role in understanding the health impacts of temperature exposure. When modeling temperature impacts using daily mean, maximum, and minimum temperatures, we observed the strongest effect of extreme high temperature when using daily minimum temperature (table 2). Nighttime heat stress can place an additional burden on the body by preventing cooling and recovery from daytime heat [54]. Thus, further research is warranted on the role of minimum temperatures in climate-related health effects, particularly to explore sleep-related mechanistic pathways.

We acknowledge our study has several limitations. First, our results are vulnerable to ecological fallacy. While we minimized this risk by using a small-area analysis approach, our findings remain at the population level and are not directly applicable to individual-level outcomes. Second, we did not adjust for air pollution; however, this is unlikely to be a confounder for temperature-mortality associations given that daily air pollution cannot affect local daily ambient temperature [55]. Third, potential effect modification not included in this analysis may have gone unrecognized. Lastly, we did not account for the potential mitigating role of air conditioning access.

Our study makes important contributions to understanding the health impacts of non-optimum temperatures in Caribbean SIDS. By preserving inter-municipality heterogeneity, we employed the innovative small-area approach to quantify the mortality burden beyond urban settlements in Puerto Rico. This approach allowed us to capture substantial intra-island temperature variability—challenging the assumption that tropical settings are uniformly warm—and to detect spatial patterns in temperature-related mortality that may be obscured in aggregated analyses. Our findings reveal great spatial heterogeneity in temperature-related deaths, underscoring the importance of implementing municipality-specific adaptations strategies. These results facilitate the identification of the most vulnerable municipalities to temperature extremes and whether cool or warm temperature pose a higher risk. This provides key insights into where to allocate resources to address climate impacts, and which interventions will be most impactful in different regions. Collectively, our findings suggest that the health impacts of temperature in the Caribbean and Latin America may be underestimated in multi-country studies that rely on single-city estimates and do not to fully capture local-scale exposure variation. Our approach may offer a more suitable framework for evaluating temperature-related health risks in tropical regions and other SIDS.

## 5. Conclusion

Our findings suggest that exposure to non-optimum temperatures is associated with increased mortality across the 78 municipalities in Puerto Rico, contributing to 3.88% of excess mortality island-wide. We observed substantial spatial heterogeneity: coastal and urban municipalities experienced greater mortality burdens from high temperatures, while rural and mid-center municipalities faced burdens from low temperatures. These results will provide critical epidemiological evidence to inform effective and target public health interventions addressing vulnerability to climate change.

## Data Availability

The data cannot be made publicly available upon publication because they contain sensitive personal information. The data that support the findings of this study are available upon reasonable request from the authors.
